# Rate and timing of return to sport following reverse total shoulder arthroplasty: a retrospective study of an active Australian cohort

**DOI:** 10.1016/j.jseint.2026.101751

**Published:** 2026-06-18

**Authors:** Jacob B. O'Brien, Navin Gurnani, Teresa M. Johnson, Mark D. Haber

**Affiliations:** a Faculty of Medicine, Health and Human Sciences, Macquarie University, Sydney, NSW, Australia; b Southern Orthopaedics, Wollongong, NSW, Australia

**Keywords:** Reverse total shoulder arthroplasty, Return to sport, Glenohumeral osteoarthritis, Rotator cuff arthropathy, Shoulder, Sport

## Abstract

**Background:**

Reverse total shoulder arthroplasty (rTSA) is increasingly being used for younger and more active patients; however, there is scarce reporting of return to sport (RTS) outcomes. This study aims to describe 12-month post-operative outcomes and the rate of RTS in an active Australian cohort undergoing rTSA and explores patient and surgical factors that may affect these results.

**Methods:**

This was a retrospective single-surgeon study of physically active patients who underwent rTSA (Equinoxe, Exactech Inc., USA) for glenohumeral osteoarthritis or rotator cuff arthropathy between January 2020 and January 2024. Twelve-month outcomes included RTS parameters (rate, timing, and level of performance), pain, and patient satisfaction with function. Multivariable regression models were used to determine any predictors or associations in outcomes.

**Results:**

One hundred fifty-two patients were included with a mean age of 70 (7.7) years, 36% female, and pre-operative participation in a wide range of sports. Patients demonstrated a RTS rate of 78.3% (119/152; 95% confidence interval: 70.7%-84.4%) with a median return time of 4 months. Overall, patients reported high satisfaction with their function (85/100) and low levels of pain (11/100). No statistically significant baseline demographic or surgical predictors of RTS were identified.

**Conclusion:**

In this selected active Australian cohort, rTSA was associated with high rates of RTS, high patient satisfaction, and low pain at 12 months, with patients returning to a wide variety of sports. Future research should continue to investigate mediators that may affect these outcomes and provide clarity on these findings.

Since its development in the 1980s, reverse total shoulder arthroplasty (rTSA) has become a reliable treatment option for many shoulder pathologies, including those suffering from glenohumeral osteoarthritis (GHOA) or rotator cuff arthropathy (RCA). Surgical advancements have led to post-operative improvements in shoulder function, pain, and quality of life and are now enabling its use in younger and more active patients.^5^^,^^6^ With this broader demographic and proven results, there has been an increasing demand and expectation among patients to return to higher level physical activity and sports following shoulder surgery. Despite the success, there has been limited research assessing outcomes related to return to sport (RTS) and higher levels of activity in rTSA cohorts. Furthermore, the current literature provides limited guidance on which sports patients may return to and the timing of post-operative return.^7^

The systematic reviews that have reported on RTS have shown that the results are variable across the cohorts and the published results may be difficult to translate into meaningful clinical predictors for patients.^7^^,^^15^^,^^16^ Most recently, the systematic review by Singh et al (2025)^23^ included 1844 rTSA patients, and across all studies, the rate of RTS ranged between 56 and 95%. Much of the primary research identified in these reviews have been of small sample size and with inconsistent reporting of RTS parameters, therefore limiting the ability of these findings to guide clinical practice. In addition, most of the research in this field reports on procedures completed in the United States, with only 1 identified report based on Australian data.^1^ This geographic distribution may also limit generalizability of findings to other countries particularly in relation to sport type, participation rates, and expectations.

Given rising sporting participation in Australia, and the diversity of sports participation that can be seen around the world,^13^ it is important to add to the current body of literature. Further evidence will support informed counseling and help set realistic patient expectations regarding RTS after rTSA.

The study aimed to determine the rate and timing of RTS following rTSA in an active Australian cohort to facilitate clinicians in counseling their patients and to help guide patient expectations. Secondary objectives were to assess 12-month pain and satisfaction outcomes and to identify patient and clinical predictors of these short-term outcomes.

## Materials and methods

### Study design and setting

This study was a single-surgeon, retrospective analysis of consecutive patients who underwent rTSA from January 2020 to January 2024. Participants were included in the study if they had undergone a primary rTSA, for the diagnosis of GHOA or RCA, and were deemed physically active for having participated in sport within 3 years prior to their surgery to determine a ‘return’ to sport rate as per the criteria in previous studies.^5^^,^^7^^,^^16^ Patients who underwent rTSA for other diagnoses, including fractures and septic arthritis, or as revision surgery were excluded, as well as those on work-cover.

### Surgical and post-operative protocol

All procedures were performed by a single fellowship-trained shoulder surgeon using a standard deltopectoral rTSA approach and a single prosthesis design (Equinoxe; Exactech Inc., Gainesville, FL, USA). Preparation and component implantation were undertaken according to the manufacturer's operative guide, and all glenospheres were lateralized to the level of the acromion. Subscapularis repair was performed at the surgeon's discretion based on tendon quality, tissue mobility, and interoperative stability. This approach reflects common practice. Patients followed a standardized rehabilitation protocol with sling immobilization, early protected passive/assisted motion, progression to active motion from 4 to 6 weeks, and strengthening at approximately 10–12 weeks.

### Data collection and outcome measures

Patient characteristics and surgical factors were collected for all included participants, including age (at time of surgery), gender, primary diagnosis, previous sports participation, dominant hand, operated side, and whether a subscapularis repair was completed. Patients were asked to complete a pre-operative and post-operative questionnaire containing the following 2 standardized patient-reported outcome measures (PROMs): Western Ontario Osteoarthritis of the Shoulder Index, and Oxford Shoulder Score. These PROMs are validated and commonly used outcome measures for shoulder surgery including rTSA.^21^

At 12 months, patients were contacted to complete a RTS questionnaire that was conducted via research electronic data capture.^12^ This questionnaire was developed similarly to surveys previously used in the literature to ask closed-ended questions on RTS parameters (Supplementary Table S1).^14^^,^^20^^,^^22^ RTS was defined as resuming at least fortnightly participation in the patient's pre-operative sport. Patients who changed to an alternative sport without returning to their pre-operative sport were not classified as having returned to sport. Patients returning less frequently than their pre-operative participation were classified as having returned to sport if they met the prespecified threshold of at least fortnightly participation. Level of performance returned to was recorded separately. Participants were able to provide responses for more than 1 sport, and a free text box was available for patients who were unable to RTS to explore the reasons for no participation. Free-text explanations were analyzed quantitatively with responses coded into nonmutually exclusive categories: shoulder-related limitation, fear of reinjury, nonshoulder medical comorbidity, lifestyle change, and practical/access barriers. Patients were also asked to score their satisfaction with their function at 12 months (0-100), pain score (visual analog scale: 0-100), and rate their difficulty with performing activities of daily living (ADLs).

### Statistical analysis

Statistical analysis was performed using Jamovi (version 2.3.28c). Descriptive data are presented as mean (standard deviation), median (interquartile range), or frequency (%), as appropriate. Continuous variables were assessed for normality using visual inspection of histograms and Q–Q plots. Comparisons were performed using Student *t*-test or Mann–Whitney *U* test for continuous variables and chi-square test for categorical variables. As this was a retrospective analysis of all eligible patients, no a priori sample size calculation was performed, and statistical significance should therefore be interpreted in the context of the available sample size. The significance level was set at *P* < .05.

For the primary outcome, RTS rate was calculated as a proportion with 95% confidence intervals (CIs) calculated using the Wilson score method. Multivariable regression models were used to evaluate associations between baseline demographics and clinical factors (age, gender, diagnosis, dominant-side surgery, and subscapularis repair) and 12-month outcomes (RTS, pain and satisfaction). These baseline variables have been previously identified as potential moderators in reverse total shoulder replacement outcomes.^1^^,^^8^^,^^20^ Model discrimination for the RTS logistic regression model was assessed using the c-statistic/area under the receiver operating characteristic curve. Linear regression assumptions were assessed using residual and Q–Q plots. Because RTS and pain were assessed at the same 12-month time point, models including both outcomes were considered exploratory and interpreted as associative rather than causal.

### Ethical aspects

This study was conducted in accordance with the ethical standards of the Macquarie University Human Research Ethics Committee, under the approved protocol (reference: 520251912063603). Clinical research governance was also attained by MQ Health Clinical Innovation Research Audit Committee (reference: MQCRG2025031). Patient confidentiality was maintained throughout the study in compliance with institutional and national guidelines.

## Results

Between January 2020 and January 2024, 361 consecutive patients underwent primary rTSA for GHOA or RCA by the senior surgeon. Of these, 152 (42.1%) reported participating in sport within 3 years prior to surgery and met inclusion criteria for this study. Patient characteristics of the included cohort are summarized in Table I with a mean age of 70 (range: 47 - 93) years. The remaining 209 patients did not participate in pre-operative sports and were therefore excluded. Compared to excluded patients, those included were more commonly male (*P* = .003), with no other significant differences identified (Supplementary Table S2).

**Table I:** Patient characteristics and pre-operative clinical details.

Characteristics	All participants (n = 152)
Gender (% female)	55 (36.2%)
Age at surgery	70.3 (7.71)
Dominant hand	
Right	144 (94.7%)
Left	6 (3.9%)
Ambidextrous	2 (1.3%)
Diagnosis	
GHOA	79 (52.0%)
RCA	73 (48.0%)
Surgery	
Side of surgery (right)	90 (59.2%)
Dominant side (yes)	94 (61.8%)
Subscapularis repair	100 (65.8%)

*GHOA*, glenohumeral osteoarthritis; *RCA*, rotator cuff arthropathy; *SD*, standard deviation.

Table describing the patient characteristics (gender, age, dominant hand) and clinical details (diagnosis, side of surgery, and when a subscapularis repair was completed) of all included patients. Data expressed as frequency n (%) and mean and standard deviation (SD).

**Figure 1:**
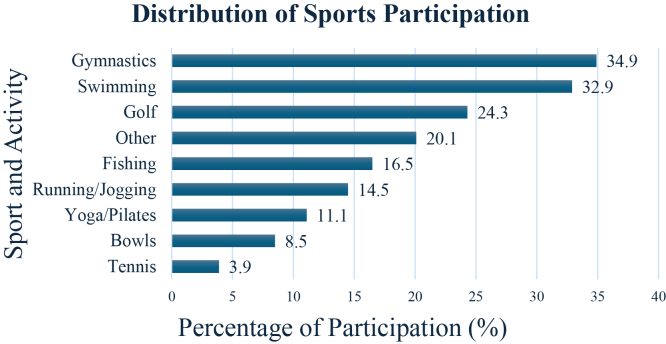
Distribution of sports participation. Horizontal bar graph demonstrating the type and frequency of sport participation pre-operatively among patients (n = 152). Participants could engage in multiple activities; percentages total more than 100%. ‘Other’ included responses that had reduced frequency of participation (ie, cycling, hiking, kayaking, dancing, and skiing). Gymnastics, swimming, and golf were identified as the most commonly participated sport.

The primary diagnosis and reason for rTSA was evenly distributed (GHOA: 52%; RCA: 48%), 61.8% had surgery on their dominant side and 65.8% had a repairable subscapularis. The distribution of pre-operative sports participation is depicted in Figure 1.

All 152 included patients completed the RTS questionnaire at 12 months. Pain and satisfaction scores were available for 146 patients (96.1%) and 12-month PROMs (Oxford Shoulder Score and Western Ontario Osteoarthritis of the Shoulder Index) were completed by 60 patients (39.5%).

At 12 months, 78.3% (119/152) had returned to sport following surgery (95% CI: 70.7% - 84.4%), with 80.6% of patients returning to a similar or improved level of performance (Table II). The median RTS time was 16 weeks (4 months) and the breakdown of return to individual sports is described in Table III. Lower impact activities such as yoga/Pilates and swimming had the highest return rates (94% and 78%, respectively), with more than 70% of these patients returning to a similar or improved level of activity. Racquet sports demonstrated the lowest return rate (33%).

**Table II:** Overall patient outcomes at 12 mo following rTSA.

Clinical outcome	All participants (n = 152)
Patient satisfaction (0-100)	84.6 (27.5)
Pain (0-100)	11.3 (19.8)
Difficulty with ADLs	
None	55.9%
Mild	31.6%
Moderate	9.6%
Severe	2.9%
Return to sport	
Rate of RTS	78.3%
Return to same or higher level	80.6%
Time to RTS∗	16.1 [11.0-26.4]

*ADLs*, activities of daily living; *RTS*, return to sport; *SD*, standard deviation; *rTSA*, reverse total shoulder arthroplasty; *IQR*, interquartile range.

Twelve-month outcomes for patient satisfaction, pain, difficulty with activities of daily living, and return to sport parameters. Data expressed as frequency (%) and mean and standard deviation (SD).

∗ Time to RTS was summarized using median [IQR].

**Table III:** Return to individual sport outcomes after rTSA.

Sport	Rate of return to sport	Return to same or higher level	Time of return (weeks)
Yoga/Pilates	16/17 (94.1%)	75.0%	17.4 [13.3-26.3]
Swimming	39/50 (78.0%)	74.3%	20.6 [12.9-33.5]
Bowls	10/13 (76.9%)	100%	12.5 [10.9-17.2]
Gymnastics	40/53 (75.5%)	92.5%	17.5 [11.1-30.3]
Fishing	18/25 (72.0%)	72.2%	20.4 [13.1-25.9]
Golf	25/37 (67.6%)	84.0%	16.9 [11.0-20.9]
Running	11/22 (50.0%)	100%	13.1 [11.3-26.9]
Tennis	2/6 (33.3%)	100%	25.9, 38.9∗

*IQR*, interquartile range; *rTSA*, reverse total shoulder arthroplasty.

Data expressed as Frequency (%) and Median [IQR].

Sports with fewer than 10 participants should be interpreted cautiously due to small sample size.

∗ Individual return times are presented for sports with n < 4 rather than summary statistics.

Among the 33 patients who did not RTS, 24 (72.7%) provided analyzable reasons. The most frequently reported reason was non–shoulder-related medical limitations (10/24, 41.7%), followed by shoulder-related pain/limitation (6/24, 25.0%). Lifestyle changes and fear of reinjury were each reported by 5 patients (20.8%), while practical or access barriers were less common (2/24, 8.3%).

Overall, this cohort reported high satisfaction with 12-month function, with a mean score of 85/100 (95% CI 80 – 89), and low pain, with a mean score of 11/100 (95% CI 8 – 15). As for patient ADLs, 87.5% of patients could perform all ADLs with either no or minor difficulty (Table II).

Baseline demographics and surgical characteristics were similar between patients who did and did not RTS (Table IV). Subscapularis repair was more common in the no-return group, although this did not reach statistical significance. Twelve-month subjective outcomes between those who returned to sport and those who did not are also described in Table IV. Pain at 12 months was significantly reduced in those who returned to sport in this unadjusted comparison (mean difference −21.8, 95% CI -29.7 – −13.9; *P* < .001) suggesting a close association with RTS status. Patient satisfaction with shoulder function at 12 months showed a trend toward those who returned to sport, although not statistically significant.

**Table IV:** Patient characteristics and 12-mo outcomes by return to sport status.

Variable	Returned to sport (n = 119)	No return to sport (n = 33)	Mean difference (95% CI)	Cohen d	*P* value
Baseline characteristics					
Age at surgery	70.50 (7.46)	69.50 (8.61)	-	-	.498
Gender (% female)	37.8%	30.3%	-	-	.427
Diagnosis					
GHOA	47.1%	51.5%	-	-	.650
RCA	52.9%	48.5%			
Surgery					
Dominant side surgery	58.8%	72.7%	-	-	.162
Subscapularis repair	62.2%	78.8%	-	-	.075
12-mo outcomes					
Pain	7.24 (14.20)	29.04 (29.91)	−21.80 (−29.68 to −13.91)	1.210	**<.001**
Satisfaction	86.41 (26.6)	77.10 (30.32)	9.27 (−2.12 to 20.66)	−0.339	.110

*GHOA*, glenohumeral osteoarthritis; *RCA*, rotator cuff arthropathy; *CI*, confidence interval; *SD*, standard deviation; *rTSA*, reverse total shoulder arthroplasty.

Table comparing the baseline characteristics and clinical details between those who did, and did not return to sport following rTSA., 12-mo outcomes are also observed with the mean difference and significance shown for pain and patient satisfaction scores.

Data expressed as frequency (%) and mean and standard deviation (SD).

Continuous variables compared with independent t-test; categorical variables with χ^2^.

Mean differences are shown only for continuous outcome measures.

Bold *P* values indicate *P* < .05.

In a multivariable logistic regression model, no statistically significant associations were identified between baseline or surgical factors and RTS (likelihood ratio χ^2^ = 6.28, df = 5, *P* = .28; McFadden's R^2^ = 0.04), and no individual predictor reached statistical significance (Supplementary Table S3). The model demonstrated modest discrimination, with a c-statistic/area under the receiver operating characteristic curve of 0.64. Given the number of nonreturn events, the multivariable RTS model may have been underpowered for small-to-moderate associations; therefore, null findings should be interpreted with caution.

In a secondary exploratory model including 12-month pain scores, higher pain was independently associated with a lower likelihood of RTS within 12 months (OR per 1-point increase 0.95, 95% CI 0.93 – 0.98; *P* < .001). A 10-point higher pain score corresponded to an approximately 38% reduction in the odds of RTS.

Multivariable analyses were completed for 12-month outcomes, pain, and satisfaction (Supplementary Table S4). Overall, most patients reported minimal pain at 12 months. Older age was statistically associated with slightly lower pain (β = −0.57 per year, 95% CI -1.02 − −0.12; *P* = .013), although the magnitude of this association was modest and its clinical significance may not be meaningful at the individual patient level. No other associations with pain were observed, and no associations with 12-month satisfaction were present in this model (all predictors *P* > .05).

There was limited response to 12-month PROMs, and patients with completed data were significantly more likely to have undergone subscapularis repair compared to those without PROMs data (93.3% vs. 47.3%, *P* < .001; Supplementary Table S5), indicating significant selection bias in collection. The PROM data were therefore considered exploratory and should not be generalized. With this limitation, statistically significant improvements in PROMs were observed at 12 months (both *P* < .001); however, these improvements should not be assumed to be representative of the full cohort (Supplementary Table S6). Both scores exceeded the minimal clinically important difference.^19^

## Discussion

The most important finding of this study was a 78.3% rate of RTS after rTSA in our active Australian cohort, with a median return time of 4 months after surgery. This high proportion is consistent with previous studies, which report up to 90% RTS rate in similar cohorts,^7^^,^^16^^,^^23^ though our return time was marginally quicker than previously reported by Franceschetti et al^7^ (5 months). This cohort was intentionally restricted to active patients and was therefore a selected active subgroup of the broader rTSA population. As such, the RTS rate reported in this study should not be interpreted as generalizable to all patients undergoing rTSA.

The study observed that this Australian cohort participated in a wide range of sporting activities. The array of sports in this cohort was broad compared to other studies around the world representing the diversity of Australian activity. In most US studies, Golf is dominant in the data, whereas in Swiss and Korean studies, hiking and swimming were most popular, respectively. It was observed that the rate and timing of RTS varied across activities, supporting the value of reporting individual sports rather than overall RTS alone. In this cohort, yoga/Pilates and swimming had the highest rate of return, whereas racquet sports had the lowest. This is broadly consistent with prior literature suggesting that lower-impact activities are more reliably resumed, while overhead racquet sports may be limited by greater shoulder demands.^5^

In this study, baseline demographics and surgical factors were not statistically significant predictors of RTS. This finding suggests that any individual effect may be modest, relative to the current sample size, or that RTS is influenced by factors not measured in the author's routine data collection. This null finding complements the observed high overall rate of RTS as it supports counseling that RTS may be achievable across a broad range of patient demographics. It also indicates that predicting individual RTS remains challenging.

In contrast to this, previous studies have reported relationships between RTS and baseline characteristics. Al Housni et al^1^ found a correlation with both male sex and younger age leading to an improved rate of RTS. However, this correlation was made on a small cohort who participated in pre-operative sport (n = 22). Garcia et al^9^ also observed that a younger age (<70 years old) was associated with an improved rate of RTS, but no other characteristics were identified as significant in the unadjusted analyses.

Brusalis et al^3^ observed a greater ability to RTS in those with a diagnosis of GHOA, in a multisurgeon registry analysis and Pennington et al^20^ displayed similar findings favoring GHOA in an active cohort of 106 patients showing GHOA was a significant predictor of RTS. It has been suggested that it is the intact rotator cuff that enables these patients to return to higher levels of activity; however, these results were not replicated in this present study, and further research is required to clarify these findings.

Through secondary analysis, this study found a significant association between lower 12-month pain and RTS status. However, as both pain and RTS were assessed at the same time point, the direction of this association cannot be determined. Lower pain may facilitate RTS, RTS may contribute to lower perceived pain, or both may reflect better overall recovery. This association raises an important area for discussion, including whether identifying and addressing contributors to persistent post-operative pain may influence RTS outcomes after rTSA.

The present study identified a modest but statistically significant association between age and 12-month pain, with younger patients reporting higher pain scores. Although the strength of this association is small, it may be explained clinically by older patients having lower activity demands, different sport profiles, or different perception of symptoms.

The role of subscapularis repair in rTSA remains debated. This study found that there was no statistically significant association between subscapularis repair and 12-month outcomes including pain, satisfaction, and RTS status. This contrasts with studies by Friedman et al^8^ and Godin et al^10^ who identified that those with a repair had significant post-operative improvement compared to no repair in PROMs and pain scores. Collin et al,^4^ however, reviewed a cohort of 86 patients after rTSA and explored functional outcomes and subscapularis repair integrity based on sonography. They found that 47% of repairs had failed at 2 years and when accounting for repair integrity, there were no significant differences in PROMs and pain. The report discussed that the conflicting findings of the role of repair may be due to whether the repair was functional and intact at the time of follow-up. Subscapularis repair status should also be interpreted cautiously because repairs were not randomized in this study or the reviewed literature. The decision for repair was based on intraoperative findings as per common practice. This introduces potential confounding by indication, whereby repairability may reflect favorable local tissue factors rather than the independent effect of repair.

Our findings suggest that factors beyond baseline demographics may drive outcomes and several other factors have been suggested in the literature that may contribute to persistent pain after rTSA and, in turn, influence results. Implant positioning has emerged as a possible factor contributing to post-operative outcomes.^2^^,^^24^ Boutsiadis et al^2^ described the effect of implant lateralization and distalization on range of motion, PROMs, and pain scores and concluded that improved outcomes were correlated with a specific window of implant positioning. Patient posture has more recently been discussed, and Moroder et al^18^ described 3 scapulothoracic phenotypes and found that differences in posture significantly impact outcomes after rTSA. On top of anatomical and structural differences, patient psychological factors have also been reviewed and patients with a history of mental health conditions have been shown to have significantly reduced post-operative functional outcomes in shoulder arthroplasty, although the reporting of this in RTS data is severely limited.^16^^,^^17^^,^^25^ Pre-operative pain catastrophising scores and ‘fear of reinjury’ have also been correlated with increased post-operative pain and reduced function after shoulder surgery.^11^^,^^26^ Future studies should continue to investigate these potential drivers of persistent post-operative pain and whether targeted strategies lead to improved function and RTS after rTSA.

Clinically, these findings may assist counseling of active patients considering rTSA. Patients can be advised that RTS is common in selected active cohorts, but expectations should be sport specific. Surgeons should highlight that while overall RTS rates are favorable, participation in overhead racquet sports may be limited, when compared to lower-impact sports.

### Limitations

This study has several limitations. Firstly, the retrospective design introduces potential bias including nonblinded post-operative assessment and recall bias associated with 12-month questionnaires. However, this approach is consistent with existing literature and enabled the use of prospectively collected data to address clinically relevant questions.

Secondly, follow-up was limited to 12 months, and outcomes may differ over longer-term follow-up. As such, longer-term studies including implant survivorship and revision or complication rates are warranted. However, the aim of the study was to focus on short-term outcomes. Despite the study's relatively large cohort, the sample size may limit the detection of smaller effects, especially with the inclusion of smaller subgroups and borderline events-per-variable in regression models that may have been underpowered to detect modest associations.

Validated pre-operative and post-operative PROMs were only available for 60 patients (39.5%) and should therefore be interpreted as exploratory. This subset was not representative of the full cohort as PROM responders were more likely to have undergone subscapularis repair, introducing selection bias and limiting interpretation and generalizability. The mechanism of this bias is unclear. The incomplete PROM data also limit direct comparison with the current literature. Additionally, objective measures (range of motion and strength) and radiographic outcomes would have strengthened the study; however, the reported measures align with patient-specific care in this active cohort.

Finally, the generalizability of these findings is limited due to the selected active subgroup included and may again be limited due to the single-surgeon, single-implant design and exclusion of other indications for rTSA, such as fractures and revision procedures.

## Conclusion

In this selected active Australian cohort, rTSA was associated with high rates of RTS, high patient satisfaction and low 12-month pain. While no statistically significant predictors of RTS were identified, reduced 12-month pain was associated with higher rates of RTS. These findings provide evidence-based guidance for patient counseling in an active population, however, may not be generalizable to the broader rTSA population. Future research should investigate longer-term outcomes and consider the role of physical and psychological confounders on RTS and function.
