# Supplementary material for: Rate and timing of return to sport following reverse total shoulder arthroplasty: a retrospective study of an active Australian cohort
Source: JSES Int. 2026 Jun 18;10(5):101751. doi: 10.1016/j.jseint.2026.101751 (PMC13427428; doi:10.1016/j.jseint.2026.101751)
Supplement: Supplementary Tables S1-S6 [file mmc1.docx]

**Supplementary Table S1: Patient Reported Return to Sport and Function Questionnaire**

Questionnaire items and response options used to assess postoperative satisfaction, pain, activities of daily living (ADL) difficulty, preoperative sport participation, return-to-sport status, time to return, and perceived level of return compared with preoperative participation.

| **Questions** | **Answer Options** |
| --- | --- |
| Q1. How satisfied are you with your shoulder and total shoulder replacement surgery? | 0 – 100  (0 = Completely unsatisfied; 100 = Completely satisfied) |
| Q2. Currently, how would you rate the pain in your shoulder? | 0 – 100  (0 = Completely no pain; 100 = Extreme pain) |
| Q3. Currently, how difficult is it to perform your normal activities of daily living? | - No difficulty - Mild difficulty - Moderate difficulty - Severe difficulty |
| Q4. Please indicate what sport you regularly (at least fortnightly) participated in prior to your surgery | - *Finite list of sports - Other: Free text option |
| Q5. Have your returned back to this sport regularly (at least fortnightly) since surgery? | - Yes - No: Free text option to indicate why no return to sport |
| Q6. When did you return to this sport post-surgery regularly? | Date: dd/mm/yyyy |
| Q7. To what level have you returned to this sport, compared to before surgery? | - Worsened - Same - Improved |

**Supplementary Table S2: Comparison of Patient Characteristics by Inclusion Status**

Baseline demographics and surgical characteristics compared between included (n = 152) and excluded (n = 209) patients. Data are presented as mean (SD) or frequency (%). Continuous variables were compared using independent t-tests and categorical variables using χ² tests; bold p-values indicate p < 0.05.

| Characteristics | Included  (n = 152) | Excluded  (n = 209) | P value |
| --- | --- | --- | --- |
| Age at surgery | 70.3 (7.71) | 71.7 (8.52) | 0.130 |
| Gender (% Female) | 36.2% | 53.6% | **0.003** |
| Diagnosis |  |  |  |
| GHOA | 48.0% | 49.1% | 0.762 |
| RCA | 52.0% | 50.9% |  |
| Surgery |  |  |  |
| Dominant Side Surgery | 61.8% | 57.3% | 0.500  0.133 |
| Subscapularis Repair | 65.8% | 73.9% |  |
| Data expressed as frequency (%) and mean and standard deviation (SD).  Continuous variables compared with independent t-test; categorical variables with χ²  Bold p values indicate p < 0.05.  Patients included were significantly more likely to be male (p = 0.003). | | | |

**Supplementary Table S3: Multivariable logistic regression for Return to Sport**

Adjusted odds ratios (ORs) with 95% confidence intervals (CIs) for predictors of return to sport following rTSA (model n = 152; returned n = 119; not returned n = 33). Outcome coded as returned to sport = 1. Model fit statistics are reported (McFadden’s pseudo-R² and global likelihood ratio test).

| Predictor | OR | 95% CI | P value |
| --- | --- | --- | --- |
| Age, per 1-year increase (Years) | 1.012 | 0.962 – 1.062 | 0.650 |
| Gender  (Female vs Male) | 1.347 | 0.571 – 3.181 | 0.496 |
| Diagnosis  (GHOA vs RCA) | 0.846 | 0.383 – 1.870 | 0.680 |
| Subscapularis Repair (Yes vs No) | 0.488 | 0.191 – 1.252 | 0.133 |
| Dominant Side Operation  (Yes vs No) | 0.521 | 0.220 – 1.242 | 0.139 |
| Multivariable logistic regression model for return to sport.  Model n = 152; returned to sport n = 119, no return n = 33. Outcome coded as “returned to sport = 1”.  Model fit: McFadden’s pseudo-R² = 0.04. Global likelihood ratio test: χ² = 6.28, df = 5, p = 0.280. Model discrimination was modest, with a c-statistic/AUC of 0.640. | | | |

**Supplementary Table S4: Multivariable Linear Regression Models for 12-Month Outcomes**

Adjusted unstandardized β coefficients (95% CIs) for predictors of 12-month pain and satisfaction in the rTSA cohort (n = 152), including age, sex, diagnosis, subscapularis repair, and dominant-side surgery. Model fit (R², adjusted R², F-test p-values) and assumption checking (residual and Q–Q plots) are summarized; bold p-values indicate p < 0.05.

| Predictor | Predictors of 12-month pain | | | Predictors of 12-month Satisfaction | | |  |
| --- | --- | --- | --- | --- | --- | --- | --- |
|  | β coefficient | 95% CI | P value | β coefficient | 95% CI | P value |  |
| Age, per 1-year increase (Years) | -0.569 | -1.021 − -0.120 | **0.013** | 0.275 | -0.336 − 0.886 | 0.375 |  |
| Gender  (Female vs Male) | 2.943 | -4.072 − 9.961 | 0.408 | -4.739 | -14.389 − 4.912 | 0.333 |  |
| Diagnosis  (GHOA vs RCA) | -3.406 | -10.183 − 3.362 | 0.321 | -0.221 | -9.518 − 9.077 | 0.963 |  |
| Subscapularis Repair  (Yes vs No) | 1.082 | -5.983 − 8.144 | 0.762 | 1.114 | -8.702 − 10.930 | 0.823 |  |
| Dominant Side Operation  (Yes vs No) | 1.402 | -5.492 − 8.293 | 0.688 | -1.232 | -10.731 − 8.267 | 0.798 |  |
| Multivariable linear regression models for cohort (n = 152) adjusted for age, sex, diagnosis, subscapularis repair and dominant side operation.  β coefficients are unstandardized adjusted mean differences: for age, per 1-year increase; for binary predictors, for the first listed category versus the reference category.  Regression assumptions were assessed using residual and Q–Q plots. Mild residual non-normality was observed for pain and satisfaction, consistent with their bounded distributions. No major violations of linear regression assumptions were identified.  Pain model: R² = 0.058, Adj-R² = 0.022, F-test p = 0.163; Satisfaction model: R² = 0.014, Adj-R² = -0.022, F-test p = 0.857  Bold p values indicate p < 0.05. | | | | | | | |

**Supplementary Table S5: Comparison of Patient Characteristics by PROM Completion Status**

Baseline demographics and surgical characteristics compared between patients with completed PROMs (n = 61) and those without PROM completion (n = 91). Data are presented as mean (SD) or frequency (%). Continuous variables were compared using independent t-tests and categorical variables using χ² tests; bold p-values indicate p < 0.05.

| Characteristics | PROMs Completed  (n = 61) | PROMs Not Completed  (n = 91) | P value |
| --- | --- | --- | --- |
| Age at surgery | 70.92 (6.96) | 69.91 (8.19) | 0.442 |
| Gender (% Female) | 27.9% | 41.8% | 0.081 |
| Diagnosis |  |  |  |
| GHOA | 42.6% | 51.6% | 0.275 |
| RCA | 57.4% | 48.4% |  |
| Surgery |  |  |  |
| Dominant Side Surgery | 59.0% | 63.7% | 0.557  **<0.001** |
| Subscapularis Repair | 93.4% | 47.3% |  |
| Data expressed as frequency (%) and mean and standard deviation (SD).  Continuous variables compared with independent t-test; categorical variables with χ²  Bold p values indicate p < 0.05.  Patients with completed PROMs were significantly more likely to have undergone subscapularis repair (p < 0.001), indicating selection bias.  PROM results should be interpreted as a non-representative subset analysis rather than generalizable to the full cohort. | | | |

**Supplementary Table S6: Difference in Patient Reported Outcome Measures at 12 Months Following RTSA**

Preoperative and 12-month Oxford Score and WOOS Index values, mean (SD), with mean differences (95% CI) and paired t-test p-values for patients with available PROMs. WOOS = Western Ontario Osteoarthritis of the Shoulder Index; bold p-values indicate p < 0.05.

| Outcome | Pre-op | Post-op | Mean Difference  (95% CI) | P value |
| --- | --- | --- | --- | --- |
| Oxford Score | 24.93 (9.54) | 39.92 (8.63) | 15.10 (11.42 - 18.74) | **<0.001** |
| WOOS Index | 37.72 (18.98) | 76.44 (23.70) | 38.73 (31.73 - 45.62) | **<0.001** |
| Data presented as mean and standard deviation (SD).  Continuous variables compared with paired t-test; bold p values indicate p < 0.05.  WOOS Index: Western Ontario Osteoarthritis of the Shoulder Index  Patients with completed PROMs were significantly more likely to have undergone subscapularis repair (p < 0.001), indicating selection bias.  PROM results should be interpreted as a non-representative subset analysis rather than generalizable to the full cohort. | | | | |
